# Safety and efficacy of platelet-rich plasma in treatment of carpal tunnel syndrome; a randomized controlled trial

**DOI:** 10.1186/s12891-018-1963-4

**Published:** 2018-02-13

**Authors:** Seyed Ahmad Raeissadat, Afshin Karimzadeh, Masoud Hashemi, Leila Bagherzadeh

**Affiliations:** 1 Physical Medicine and Rehabilitation Research Center, Clinical Research Development Center of Shahid Modarres Hospital, Shahid Beheshti University of Medical Sciences, Tehran, Iran; 2 Physical Medicine & Rehabilitation department, Imam Hosein hospital, Shahid Beheshti University of Medical Sciences, Tehran, Iran; 3 Anesthesiology Research Center, Shahid beheshti university of medical sciences, Tehran, Iran; 4 Physical Medicine and Rehabilitation Research Center, Shahid Beheshti University of Medical Sciences, Tehran, Iran

**Keywords:** Carpal tunnel syndrome, Platelet-rich plasma, Wrist splint, Clinical trial

## Abstract

**Background:**

Carpal tunnel syndrome is the most common peripheral entrapment neuropathy, for which conservative treatments are the first measures taken. However, these measures are not usually sufficient. Recently major attention has been drawn to platelet-rich plasma for its possible effects on axon regeneration and neurological recovery. Although few studies have evaluated the effects of this treatment in carpal tunnel syndrome, further investigation is required to reach concrete conclusion.

**Methods:**

In this randomized controlled trial, women referring to the physical medicine and rehabilitation clinic at Shahid Modarres Hospital during 2016 with a diagnosis of mild and moderate idiopathic carpal tunnel syndrome were chosen. They were randomly assigned to two groups: (i) a control group using only a wrist splint, and (ii) a platelet-rich plasma group that received wrist splints along with a single local injection of platelet-rich plasma. The outcome measures were assessed via Visual Analogue Scale, the Boston Carpal Tunnel Syndrome Questionnaire and electrophysiological findings including the peak latency of sensory nerve action potential and the onset latency of the compound muscle action potential.

**Results:**

A total of 41 women were included (20 wrists as control group) and (21 wrists as platelet-rich plasma group). Before treatment there were no significant differences between the two groups except for the median peak latency of sensory nerve action potential which was significantly higher among the patients in the platelet-rich plasma group (*p* = 0.03). All the measured variables significantly decreased in both groups after 10 weeks of treatment except for the median onset latency of the compound muscle action potential (*p* = 0.472). Finally, the changes in neither of the evaluated outcome measures were found to significantly differ between the two groups, even when the analyses were adjusted for age of the patients.

**Conclusion:**

The findings of this study showed that in a relatively short period of time after treatment, a single injection of platelet-rich plasma in the wrist does not significantly add to the effects of conservative treatment with wrist splints, in regards to the women pain and symptom severity, functional status and electrophysiological parameters.

**Trial registration:**

The trial has been retrospectively registered with an ID: IRCT2017041513442N13 (Date of registration: 2017–06-19).

## Background

As the most common peripheral entrapment neuropathy, carpal tunnel syndrome (CTS) accounts for approximately 90% of cases [1]. Existing evidence based treatments for carpal tunnel syndrome, splinting, corticosteroid injection and surgery, are not 100% effective and alternative treatments are worth exploring [2–11].

Platelet-rich plasma (PRP) is an autologous biologic product of concentrated platelets, the main constituent of which is thought to be degradation products that include multiple growth factors, well known to be effective on inflammation and wound healing. Some of these factors that are identified within the alpha granules of platelets include Transforming Growth Factor (TGF), Vascular Endothelial Growth Factor (VEGF), Platelet-Derived Growth Factor (PDGF), Epidermal Growth Factor (EGF) and the Insulin-like Growth Factor-1 (IGF-1) [12].

In the last three decades, PRP has been used as a safe treatment in different settings [13, 14]. This product has recently been shown to have positive effects on axon regeneration and neurological recovery [15–22]. It has also been shown to have acceptable success rates in treatment of clinical peripheral neuropathies [23–28]. Although few of these surveys have evaluated the effects of PRP in treatment of CTS, further investigation is required to provide robust evidence on the basis of which, guidelines could be established for the application of this treatment in CTS patients. Accordingly, we aimed to compare the effects of PRP to that of wrist splinting in patients diagnosed with CTS.

## Methods

### Study design

This randomized control trial was carried out at Shahid Modarres Hospital during 2016. [29] and it was retrospectively registered at www.irct.ir (ID: IRCT2017041513442N13).

### Inclusion and exclusion criteria

Women aged between 20 to 60 years referring to the physical medicine and rehabilitation clinics with signs and symptoms of CTS and a confirmed diagnosis of mild and moderate CTS based on history (paresthesia or dysesthesia and painful swelling of the hand with clumsiness due to weakness that is exacerbated by repetitive use or sleep and improved by shaking the hand), physical examination (sensory loss and numbness in the areas of the hand, innervated by the Median Nerve (MN), positive Phalen’s test and/or Tinel’s test) and electrophysiological studies, were included as the sample population. The severity of CTS was determined according to the electrophysiological classification proposed by *Stevens* et al. [30]. Mild CTS was defined as sensory latency of longer than 3.6 ms with normal motor latency (≤4.2 ms) and moderate CTS was defined as sensory latency of longer than 3.6 ms plus a prolonged motor latency (4.3–6 ms).

On the other hand bilateral CTS, pregnancy, history of underlying metabolic diseases (such as diabetes mellitus, thyroid diseases, rheumatoid arthritis and etc.), history of local corticosteroid injection in the past 3 months, atrophy of thenar muscles, previous carpal tunnel release surgery and evidence of concomitant neuropathy or radiculopathy were considered as the exclusion criteria. Patients with PRP contraindications including history of malignancies, autoimmune or hematologic disorders, NSAID consumption 2 days prior to injection, treatment with antiplatelet and anticoagulant agents, Hb level under 12 g/dl, and platelet count under 150,000 in ml were also excluded.

### Ethical considerations

The study protocol was thoroughly explained to the patients and they were reassured that their data will be only accessible by the main researchers, will be used anonymously and that they can withdraw from the study at their will. The treatment carried low risk of adverse effects and the patients were advised to contact the physician in case of experiencing infection or fibrosis at the site of injection, continued pain and swelling or any related neuromuscular complications.

An informed written consent was obtained from the enrolled subjects. The study protocol was evaluated and approved by the institutional review board of Shahid Beheshti University of Medical Sciences and the trial was conducted according to the Declaration of Helsinki’s principles.

### Sample size calculation

To calculate sample size, change in VAS (ΔVAS) was considered as the main outcome.

Based on previous studies, we considered SD (Standard Deviation) of ΔVAS as 1.5; ΔVAS in control group as 1 and ΔVAS in treat group as 2.3.The type one error (α) was set as 95% and type 2 error (β) as 80%. Using the formula for comparing two means, the sample size was calculated as 21 in each group.

### Randomization and enrolment

**Fig. 1:**
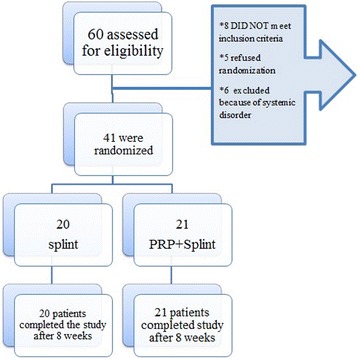
CONSORT flow chart

Patients were randomly allocated to one of two groups: (1) PRP plus wrist splint or (2) wrist splint, using an online randomization website [30] (https://www.randomizer.org). To conceal the randomization sequence, we used sequentially numbered, opaque sealed envelope (SNOSE) method. After inclusion of a patient, a physiatrist drew an envelope and opened it [31]. Figure 1 depicts the study flow diagram.

### Interventions

Patients in the wrist splint group received a prefabricated wrist splint at 5-degree wrist extension and they were instructed to put on the splint overnight for 8 weeks.

Subjects in the PRP group also received wrist splints and were instructed to use it similar to the control group. They were also treated with a single local injection of leukocyte-poor PRP, processed using the Rooyagen kit (made by Arya Mabna Tashkis Corporation, RN:312,569). Since few studies are conducted on the treatment of CTS with PRP injection, it is not clear how much PRP should be administered for these patients to yield the best response. Since corticosteroids are routinely injected in CTS patients at a volume of 1 ml and to minimize the possibility of a flare reaction along with the associated pain and discomfort following PRP injection, we decided to administer 1 ml of PRP for our patients.

For preparing 1 mL of PRP, 10 mL of blood was initially drawn from the patient’s upper limb cubital vein using an 18G needle. Then, 1 mL of ACD-A was added to the sample as an anticoagulant and passed two stages of centrifugation; first at 1600 rpm for 12 min to separate the erythrocytes and then at 3500 rpm for 7 min in order to concentrate the platelets. The resulting PRP contained leukocytes with mean concentrations of 5–10%. Eventually, PRP quantification was performed to confirm that the platelet concentration has reached 4–6 times that of the whole blood, since some studies have shown a positive effect of PRP at this concentration in musculoskeletal diseases and others have indicated that concentrations higher than 8 times can negatively affect the repair process and inhibit cellular proliferation [32, 33].

A 0.5 ml injection of lidocaine was administered via a 25G needle, inserted at the distal carpal skin crease ulnar side to the palmaris longus tendon. With the needle kept steady at the mentioned site, the syringe was changed and 0.8–1 ml of the prepared PRP was injected. Right after injection, the patients were asked to score their pain severity on a scale of 0 to 10 based on the Visual Analogue Scale (VAS). Patients were then observed in the ward for 30 min and prior to discharge, were educated about activity restrictions, probable side effects and the method of icing on the injection site. Patients’ wrists were immobilized by splints and they were administered acetaminophen codeine to use as needed for the pain. All the measured variables were assessed for both groups after 10 weeks of treatment .

### Measured parameters

Collected information included age, duration of symptoms, pain severity, electrophysiological findings, and the symptom severity and functional status of the subjects were gathered by one of the researchers. The pain severity was determined by the patients, on a scale of 0 (no pain) to 10 (agonizing pain) according to the VAS.

Electrophysiological parameters including the peak latency (PL) of sensory nerve action potential (SNAP) and the onset latency (OL) of the compound muscle action potential (CMAP), were measured for all patients using a Caldwell Sierra® Wave electromyography device by the same physician with the same settings.

The Farsi translation of Boston Carpal Tunnel Syndrome Questionnaire (BCTQ), whose validity and reliability had been assessed and confirmed by Rezazadeh et al. [34], was used for evaluating the severity of symptoms and functional status of patients.

### Statistical analysis

SPSS software for Windows v.22 (IBM Corp., Chicago, IL, USA) was used for data analysis [35]. Qualitative variables were presented as frequency and percentage and quantitative variables were calculated as mean and standard deviation. Considering the normal distribution of change variables according to Shapiro-Wilk normality tests, parametric paired t-test was used to compare the changes in each variable within one group in different follow ups. Chi-square test was also used to compare qualitative variables between the two groups. In order to control for the effects of age when comparing the change variables between the two groups, ANCOVA test was run. A *p* value of less than 0.05 was considered as statistically significant in all analyses.

## Results

All the 41 women enrolled, completed the study and eventually 20 wrists in the control group and 21 wrists in the PRP group were analyzed. Tables 1 and 2 present the findings of the comparisons between the two groups regarding qualitative and quantitative variables at the beginning of the survey.

**Table 1:** The comparison of qualitative variables between control and treatment groups at the beginning of study

Variables	Groups	Number	Percent	P Value
Side ratio (RT/Total)	Splint	20	66	0.53
	PRP+ Splint	21	55	
Dominant Hand	Splint	20	70	0.52
	PRP+ Splint	21	57	
Severity (mild/total)	Splint	20	75	0.29
	PRP+ Splint	21	60

**Table 2:** The comparison of quantitative variables between control and treatment groups at the beginning of study

Variables	Group	Number	Mean	SD	P Value	Power
Age	Splint	20	47.23	7.11	0.06	0.419
	PRP+ Splint	21	51.20	9.82		
Duration	Splint	20	14.13	8.55	0.09	0.064
	PRP+ Splint	21	13.74	11.5		
VAS	Splint	20	6.24	1.17	0.28	0.443
	PRP+ Splint	21	6.82	1.24		
Median SNAP PL	Splint	20	4.05	0.22	0.03	0.462
	PRP+ Splint	21	4.25	0.52		
Median CMAP OL	Splint	20	4.06	0.55	0.24	0.107
	PRP+ Splint	21	4.13	0.53		
SSS	Splint	20	2.73	0.40	0.58	0.475
	PRP+ Splint	21	2.43	0.73		
FS	Splint	20	2.54	0.62	0.16	0.349
	PRP+ Splint	21	2.36	0.83		

The average score of injection pain severity, based on VAS, was calculated to be 4.4 ± 2.1 with a minimum of 2 and a maximum of 8. As for the complications of PRP injection, 4 patients reported pruritus, one experienced pain in the fingers and one reported a burning sensation. The rest of patients had no side effects after injection.

Tables 3 and 4 demonstrate the changes in outcome measures after treatment in the control and PRP groups, respectively.

**Table 3:** The comparison of variables in the control group (splint) at the beginning and end of study

Variables	Group	Number	Mean	SD	P value
VAS	Before	20	6.24	1.13	< 0.001
	After	20	3.52	2.02	
MedSNAP PL	Before	20	4.05	0.25	0.001
	After	20	3.75	0.35	
MedCMAP OL	Before	20	4.06	0.59	0.002
	After	20	4.07	0.55	
SSS	Before	20	2.76	0.40	< 0.001
	After	20	1.90	0.42	
FS	Before	20	2.54	0.63	< 0.001
	After	20	1.82	0.42

**Table 4:** The comparison of variables in the treatment group (PRP + splint) at the beginning and end of study

Variables	Group	Number	Mean	SD	P value
VAS	Before	21	6.82	1.24	< 0.001
	After	21	4.02	1.92	
MedSNAP PL	Before	21	4.25	0.52	0.005
	After	21	4.12	0.63	
MedCMAP OL	Before	21	4.13	0.53	0.472
	After	21	4.15	0.52	
SSS	Before	21	2.43	0.73	< 0.001
	After	21	1.72	0.52	
FSS	Before	21	2.36	0.83	0.003
	After	21	1.83	0.73	

Table 5 presents the changes in the severity of CTS in the two groups after treatment.

**Table 5:** Changes in the severity of CTS in the two groups after treatment

Group	Condition	Normal N(%)	Mild N(%)	Moderate N(%)
Control	Before	0(0%)	15 (75%)	5 (25%)
	After	0(0%)	16 (80%)	14 (20%)
Treat	Before	0(0%)	12 (57%)	9(43%)
	After	3(14%)	10(48%)	8 (38%)

As the final comparison, the changes in each variable were compared between the two groups, the results of which are presented in Table 6. As can be seen, the changes in neither of the outcome measures evaluated were found to significantly differ between the two groups, even when the analyses were adjusted for age of the patients.

**Table 6:** The comparison of score changes in the two control and treatment groups

Variables	Group	Number	Mean	SD	P Value (Univariate)	Power	P Value (Multivariate-adjusted for age)
VAS change	Control	20	−2.90	2.1	0.845	0.073	0.398
	Treat	21	−2.76	2.4			
Median SNAP PL change	Control	20	−0.15	0.2	0.820	0.091	0.858
	Treat	20	−0.17	0.2			
Median CMAP OL change	Control	20	−0.09	0.1	0.410	0.253	0.198
	Treat	20	−0.04	0.2			
Symptom severity scale change	Control	20	−0.70	0.3	0.922	0.063	0.629
	Treat	19	−0.72	0.7			
Functional scale change	Control	20	−0.86	0.5	0.289	0.283	0.554
	Treat	19	−0.63	0.8			
Severity change	Control	20	−0.05	0.2	0.174	0.393	0.194
	Treat	21	−0.19	0.4			

## Discussion

To the best of our knowledge, this is the third controlled randomized clinical trial conducted on the efficacy of PRP injection in patients diagnosed with mild and moderate CTS. Significant improvements were observed in pain and symptom severity and functional status of patients, assessed according to the VAS and BCTQ and also electrophysiological parameters, in both PRP and splint groups, except for the median CMAP OL in the PRP group. However, the differences between the two groups of patients were not statistically significant and the PRP injection did not add considerably to the effects of wrist splint.

The idea of using PRP in the treatment of this peripheral entrapment neuropathy originated from the various experimental studies that had reported positive effects of PRP on regeneration of peripheral nerves without considerable safety risks in different settings [15–22]. Previous methodologically flawed study by Malahias 2015 has suggested a possible beneficial effect of PRP in CTS. Our randomised controlled study fails to corroborate this [26].

In another study, Uzun et al. aimed to compare the efficacy of PRP to that of corticosteroid injection in treatment of patients with CTS. [27]. The results of this study were incongruent with our findings as we did not observe any significant differences between the two groups. Although the settings of the studies differ considerably as their control group had been injected by corticosteroids while ours only used wrist splints, but still it is expected to see some similarities. However, the fact that we only followed our patients for 10 weeks could have contributed to the discrepancies observed, since the time needed for the effects of PRP injection to appear is still not established. Another difference between the two surveys that could have contributed to the incompatibility between their results was that Uzun et al. injected 2 ml of PRP for their patients, while we administered 1 ml of this concentrate.

In the most recent study, Wu et al. found results favoring PRP plus splinting over splinting alone but only at 6 months follow-up, results at shorter follow-up intervals being similar to our own. It is therefore possible that PRP may exhibit a delayed effect [28].

Accordingly, the short follow up period could be considered as the most important limitation of the present study, as it might have missed the effects of PRP injection that were probably going to appear later. Not including a placebo injection group in our study due to the limited resources was another issue that deprived us from conducting a blinded trial; however, since our findings were negative it seems unlikely to have generated a type 1 error. The small sample population is another limitation that should be addressed.

## Conclusion

A single injection of PRP in the wrist did not add significantly to the benefit of wrist splinting at 10 weeks follow up. Higher quality randomized placebo controlled trials are required to determine if this proposed new treatment is effective.
